# Dynamics of Microglia Activation in the Ischemic Brain: Implications for Myelin Repair and Functional Recovery

**DOI:** 10.3389/fncel.2022.950819

**Published:** 2022-07-11

**Authors:** Stefano Raffaele, Marta Fumagalli

**Affiliations:** Department of Pharmacological and Biomolecular Sciences, Università degli Studi di Milano, Milan, Italy

**Keywords:** microglia, oligodendrocytes, remyelination, stroke, neuroinflammation, extracellular vesicles

## Abstract

Ischemic stroke is a neurological disorder representing a leading cause of death and permanent disability world-wide, for which effective regenerative treatments are missing. Oligodendrocyte degeneration and consequent myelin disruption are considered major contributing factors to stroke-associated neurological deficits. Therefore, fostering myelin reconstruction by oligodendrocyte precursor cells (OPCs) has emerged as a promising therapeutic approach to enhance functional recovery in stroke patients. A pivotal role in regulating remyelination is played by microglia, the resident immune cells of the brain. Early after stroke, microglial cells exert beneficial functions, promoting OPC recruitment toward the ischemic lesion and preserving myelin integrity. However, the protective features of microglia are lost during disease progression, contributing to remyelination failure. Unveiling the mechanisms driving the pro-remyelination properties of microglia may provide important opportunities for both reducing myelin damage and promoting its regeneration. Here, we summarize recent evidence describing microglia activation kinetics in experimental models of ischemic injury, focusing on the contribution of these innate immune cells to myelin damage and repair. Some molecular signals regulating the pro-regenerative functions of microglia after stroke have been highlighted to provide new possible therapeutic targets involved in the protective functions of these cells. Finally, we analyzed the impact of microglia-to-OPCs communication *via* extracellular vesicles on post-stroke remyelination and functional recovery. The results collected in this review underline the importance of supporting the pro-remyelination functions of microglial cells after stroke.

## Introduction

Ischemic stroke is a neurological disorder caused by the interruption of the blood supply to a given region of the brain, mainly due to the occlusion of an afferent artery by an embolus or local thrombosis. It represents the second cause of death in Western countries and is a leading reason for long-term disability (Campbell et al., 2019; Virani et al., 2021).

The therapeutic options currently available for stroke treatment are limited to thrombolytic strategies aiming at restoring blood perfusion in the injured area. Such treatments effectively reduce the death rate in patients but have no impact on the repair of ischemic lesions, leaving a significant portion of patients with lifelong motor and cognitive disability. On this basis, the development of regenerative strategies able to restore proper brain functions, limiting stroke-induced functional deficits, represents an urgent and still unmet medical need (Campbell et al., 2019; Barthels and Das, 2020).

The ischemic pathology affects not only neurons but also oligodendrocytes (OLs), the myelin-forming glial cells of the central nervous system (CNS; Dewar et al., 2003). Following cerebral ischemia, OLs are rapidly damaged by the hyperactivation of glutamate and purinergic receptors, oxidative stress, and the impairment of mitochondrial function. These events lead to demyelination, namely the disruption of the myelin sheath, leaving axons denuded and contributing to stroke-associated functional deficits (Mifsud et al., 2014).

Recent studies also focused on the dualism of the inflammatory process following the ischemic event. Post-ischemic inflammation is mainly mediated by the activation of microglia, the brain-resident innate immune cells (Fumagalli et al., 2015), which is initially triggered by the release of damage-associated molecular patterns (DAMPs) from necrotic cells (Anrather and Iadecola, 2016). Classically, microglial activation has been associated with worse neurological outcomes after stroke by promoting ischemic secondary damage. Nevertheless, a beneficial role of these cells in limiting brain damage and supporting regenerative processes has been also described, suggesting that the response of microglia to ischemic injury is highly plastic and dynamic (Lambertsen et al., 2019).

On this basis, demyelination and neuroinflammation emerge as key mechanisms contributing to the progression of ischemic damage and neurological disability. Here, we summarize recent data describing microglial functions after stroke, focusing on the contribution of these innate immune cells to myelin damage and repair to identify promising therapeutic approaches able to promote remyelination by modulating the neuroinflammatory process.

## Oligodendrocytes and Remyelination After Stroke

During an ischemic event, the lack of oxygen leads to OL death followed by demyelination (Jia et al., 2019). Since myelin, the lipid structure that enwraps axons, is essential for the conduction of the electrical impulse and for the trophic support of neurons, its loss significantly contributes to long-term sensory-motor and cognitive deficits (Shi et al., 2015).

Post-mortem analyses carried out on brain tissue samples from stroke patients revealed that the disruption of white matter architecture was one of the main characteristic signs of cerebral ischemia (Marin and Carmichael, 2018). Indeed, myelinating OLs are very vulnerable to ischemia, due to their high susceptibility to oxidative stress, excitotoxic damage, and inflammatory cytokines (Waly et al., 2014; Guo et al., 2021b; Khawaja et al., 2021). Accordingly, the swelling and vacuolation of the OLs appeared within 3 h after middle cerebral artery occlusion (MCAo), followed by process retraction and cell death within 24 h (Pantoni, 2006; McIver et al., 2010).

Seminal studies performed on autoptic samples from multiple sclerosis (MS) patients showed that demyelination can be followed by a spontaneous repair process of the myelin sheath called remyelination, namely the formation of a new myelin sheath around denuded axons (Patrikios et al., 2006; Franklin and Ffrench-Constant, 2017). Accordingly, *in vivo* studies performed using rodent models of cerebral ischemia have shown that, a few days after stroke, the number of OLs in the areas surrounding the ischemic lesion is increased, suggesting that demyelinated axons could be remyelinated by newly-formed myelinating cells (Dewar et al., 2003; Bonfanti et al., 2017).

**Figure 1:**
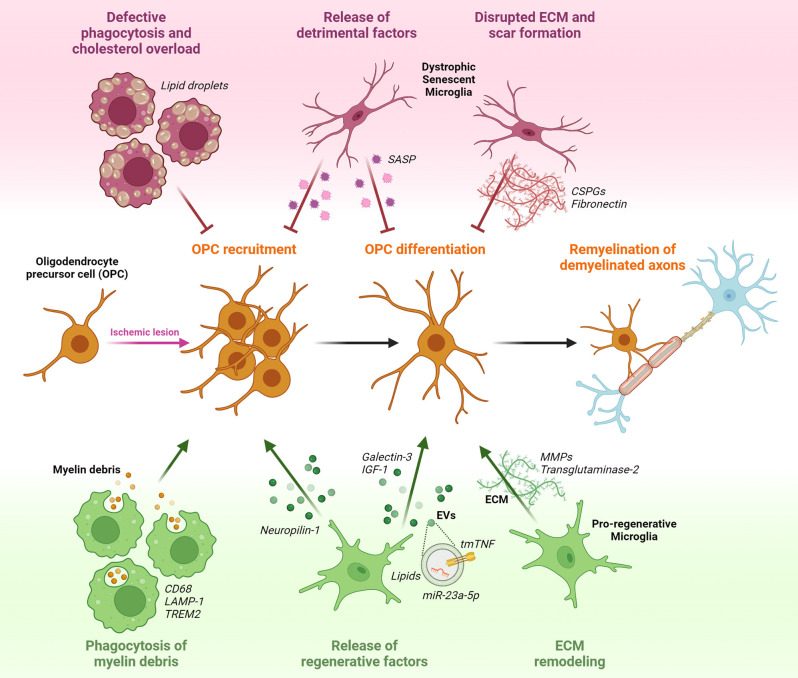
Potential effects of microglia on oligodendrocyte precursor cells (OPCs) response during post-stroke remyelination. OPCs that are present in a quiescent state within the adult brain parenchyma, respond to ischemia-induced myelin injury by increasing their proliferation rate and migratory capacity to accumulate at the lesion site. Following recruitment, OPCs start to differentiate in the attempt to replace degenerating oligodendrocytes (OLs) with newly formed myelinating cells, and to reconstitute the functional myelin sheath around denuded axons. Pro-regenerative microglia sustain remyelination by phagocytosing myelin debris, releasing regenerative factors in soluble form (Neuropilin-1, Galectin-3, IGF-1) or encapsulated into extracellular vesicles (EVs; tmTNF, lipids, miR-23a-5p), and modulating extracellular matrix (ECM) composition *via* matrix metalloproteinases (MMPs) and transglutaminase-2. Conversely, dysfunctional senescent microglia, characterized by defective phagocytosis and accumulation of lipid droplets, release detrimental pro-inflammatory factors typical of the senescence associated secretory phenotype (SASP), and favoring the deposition of chondroitin-sulfate proteoglycans (CSPGs) and fibronectin in the ECM, hamper OPC recruitment and maturation.

The main steps of the remyelination process have been well defined (Figure 1). In response to damage, remyelination begins with the transition of oligodendrocyte precursor cells (OPCs) in the vicinity of the lesion from a quiescent state to a regenerative phenotype (Moyon et al., 2015). This allows OPCs to populate and expand within the injured area through a combination of proliferation and migration; finally, they undergo differentiation, a process that culminates in the formation of the new myelin sheath (Franklin and Ffrench-Constant, 2017).

Remyelination has a neuroprotective role, as it limits axonal degeneration that follows demyelination. Thus, promoting remyelination after cerebral ischemia can be a promising therapeutic strategy to improve functional recovery (Plemel et al., 2014). The discovery that parenchymal OPCs are recruited and proliferate in the ischemic penumbra suggests the possibility of repairing ischemic lesions by implementing endogenous spontaneous remyelination mediated by these cells (Zhang et al., 2013). This evidence indicates that the manipulation of OPCs could be a promising therapeutic strategy to enhance the endogenous mechanisms of remyelination and repair (Fumagalli et al., 2016). However, it has been proven that remyelination efficiency progressively decreases with advancing age or in certain pathological conditions. This decline may be due to a defect in the recruitment of OPCs or in their differentiation capability caused by the lack of pro-regenerative factors and the presence of inhibiting cues (Gruchot et al., 2019; Skaper, 2019). Microglia, activated by myelin and neuronal injury, represent the main source of factors that influence the rapid proliferative response of OPCs, their migration toward the demyelinating lesion, and subsequent maturation (Franklin and Ffrench-Constant, 2017; Miron, 2017). For instance, during white matter development and after acute demyelination, microglia were shown to enhance OPC proliferation by increasing the expression of neuropilin-1, which in turn cross-activated PDGFRα (Sherafat et al., 2021).

This suggests that not only OL-intrinsic regulators but also the surrounding microenvironment plays a fundamental role in remyelination success (Figure 1).

## Dynamics of Microglia Activation After Stroke

Microglia are the resident immune cells of the CNS, representing about 10%–20% of all glial cells. Unlike all the other cells of the CNS, that are of ectodermal embryonic derivation, microglia derive from primitive mesodermal progenitors of the myeloid lineage and migrate in the developing CNS at an early stage of embryogenesis (Ginhoux et al., 2010).

As cells of the innate immune system, microglia monitor the surrounding environment to check for signs of damage, such as DAMPs. To this aim, under physiological conditions, microglia have a peculiar morphology characterized by a small cell body with very thin and mobile processes (Kettenmann et al., 2013). This state of restless movement allows them to continuously examine the surrounding extracellular space and interact with neighboring cells and blood vessels (Bernier et al., 2019; Cserép et al., 2020; Bisht et al., 2021).

After encountering damage signals in the CNS microenvironment, microglia undergo morphological transformations, characterized by cell body hypertrophy and retraction of cell processes, and rapidly respond by inducing specific genes, necessary to initiate an inflammatory process with the aim of containing damage evolution and favoring repair (Prinz et al., 2019). In the past, two opposite activation states of microglia have been hypothesized, an M1 pro-inflammatory and an M2 pro-regenerative phenotype. This dualistic classification has been questioned and recently replaced by a new proposal that includes numerous plastic and three-dimensional functional phenotypes (Ransohoff, 2016; Amici et al., 2017; Stratoulias et al., 2019). Accordingly, recent single-cell transcriptomic studies identified several distinct subclusters of microglia in the ischemic brain, none of which fully recapitulate classical M1/M2 signatures (Guo et al., 2021a; Li et al., 2022a; Zheng et al., 2022). On this basis, to dissect the complexity of microglia activation after stroke, it may be more appropriate to refer to specific functional features of these cells (i.e., morphology, phagocytosis, secretory pattern) rather than using this oversimplified paradigm.

**Figure 2:**
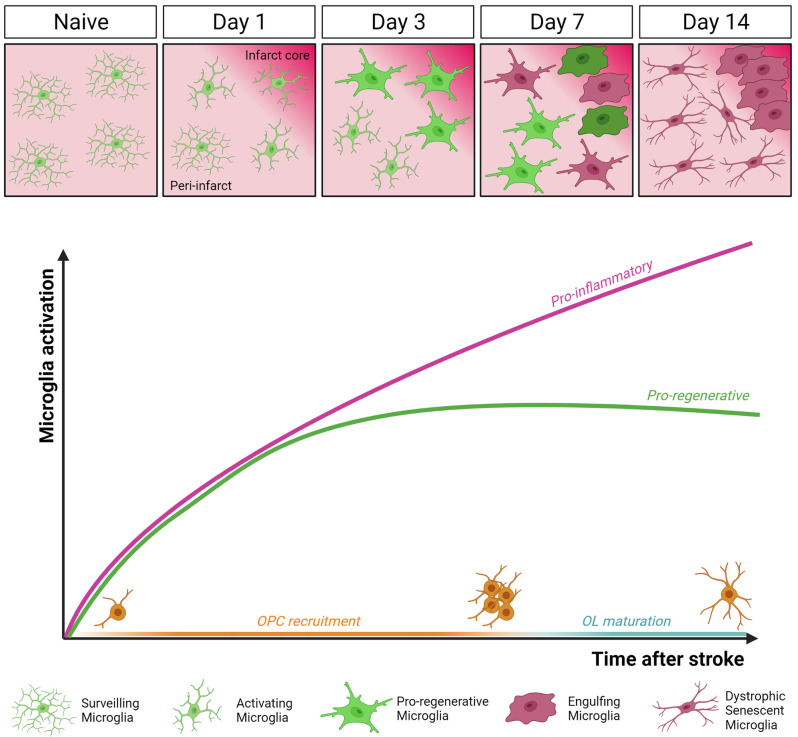
Dynamics of microglia activation after stroke. In the intact brain (Naïve), microglia display highly ramified morphology and fast process motility to surveil the surrounding tissue, as sentinels for danger signals. This allows microglial cells to rapidly respond to an ischemic event by undergoing profound morphological, molecular, and functional modifications. Two distinct temporal windows in microglial response following stroke have been defined. In the early phase (day 3 post-MCAo), microglial cells accumulating at the border of the ischemic lesion appear ameboid and hypertrophic, express both pro-inflammatory and pro-regenerative markers, and have high phagocytic capacity. At this stage, microglia were found to actively contribute to remyelination by favoring oligodendrocyte precursor cells (OPCs) recruitment and preserving myelin integrity (Raffaele et al., 2021). Conversely, at late stage after stroke (day 14 post-MCAo), dystrophic, senescent-like, pro-inflammatory microglia dominate the peri-infarct area, hindering oligodendrocyte (OL) maturation and efficient remyelination (Raffaele et al., 2021).

In the context of cerebral ischemia, microglia are promptly activated and exert both beneficial and harmful effects, depending on the specific stage of ischemic damage progression (Hu et al., 2012; Figure 2). In the MCAo model, it has been shown that microglia rapidly change their morphology and migrate to the lesion site within the first 3 days after stroke, with their number increasing up to 14 days post-MCAo (Raffaele et al., 2021). Once recruited at lesion boundaries, microglia increase the expression of pro-inflammatory genes (Yenari et al., 2010), but at the same time, they also contribute to tissue repair and remodeling by removing debris and producing anti-inflammatory cytokines, pro-angiogenic factors, and growth factors (Ponomarev et al., 2013; Ma et al., 2017).

The balance between microglial opposing functions dynamically evolves during disease progression. At early stages after ischemic injury, microglia were shown to exert protective functions, by containing detrimental astrocyte activation (Jin et al., 2017), limiting cytotoxic neutrophil infiltration within the lesion (Otxoa-de-Amezaga et al., 2019), and reducing excitotoxic injury to neurons (Szalay et al., 2016). Moreover, a beneficial microglial phenotype, characterized by the simultaneous expression of the inflammatory marker CD16/32 and of the regenerative marker YM1, has been described early after stroke, promoting the recruitment of OPCs toward the ischemic lesion and preserving myelin integrity (Raffaele et al., 2021).

On the contrary, at later time points, microglia were found to acquire a detrimental phenotype hindering brain repair (Hu et al., 2012; Rajan et al., 2019). Accordingly, at later stages after ischemia, prolonged overstimulation by chronic inflammation was shown to induce microglia immunosenescence, similar to that observed in chronic neurodegenerative diseases and aging (Raffaele et al., 2021). In particular, senescent microglia has been associated with the acquisition of a dystrophic morphology, reduced process motility, and impaired capacity of exerting pro-regenerative and neuroprotective functions, including phagocytosis and remyelination support (Rawji et al., 2016, 2020; Savage et al., 2019). These data suggest that to promote structural and functional recovery at late stages after stroke, therapeutic approaches should be aimed at restoring microglial regenerative properties rather than simply suppressing their overall activation, which inevitably leads to the loss of their beneficial effects (Fumagalli et al., 2018).

## Microglial Functions That Support Remyelination

The acquisition of a pro-regenerative phenotype by microglia represents a rate-limiting step in remyelination (Miron et al., 2013). Recent studies have indeed shown that microglia can contribute to the remyelination process by creating an environment that supports the recruitment of OPCs and their subsequent differentiation into mature myelinating OLs (Figure 1).

The first step for efficient remyelination is the clearance of myelin debris from the demyelinated area, setting the basis for subsequent OPC recruitment and differentiation (Lampron et al., 2015). To fulfill this function, microglia express genes related to phagocytosis and lysosomal pathways, including the scavenger receptor CD68 and lysosomal-associated membrane protein-1 (LAMP-1), and gain the ability of engulfing myelin debris. This capacity was shown to decrease with age, reducing remyelination efficiency (Safaiyan et al., 2016; Cantuti-Castelvetri et al., 2018). Recent studies identified several microglial receptors regulating myelin clearance capacity after stroke, as reviewed in Chen et al. (2022), including triggering receptor expressed on myeloid cells 2 (TREM2). In TREM2 knockout mice, myelin clearance was found to be impaired, leading to the accumulation of myelin debris. This in turn was associated with decreased OPC recruitment, prolonged demyelination, and axonal degeneration (Cantoni et al., 2015; Poliani et al., 2015; Gouna et al., 2021). Of note, TREM2 stimulation using an agonistic antibody rescued such defect, improving OL differentiation and remyelination (Cignarella et al., 2020). After internalization, myelin debris should be degraded and recycled to avoid saturation of the lysosomal compartment and subsequent cholesterol overload in microglia, compromising their pro-remyelination properties (Cantuti-Castelvetri et al., 2018; Bosch-Queralt et al., 2021). Therefore, genes involved in cholesterol metabolism and recycling, like the cholesterol carrier ApoE, the receptor LXRα, and the efflux transporters Abca1 and Abcg1, assume great importance for the clearance capacity of microglia around demyelinated lesions and may represent attractive therapeutic targets (Bosch-Queralt et al., 2021).

Inflammation-induced release of soluble factors by microglia is another important aspect affecting remyelination, as indicated by several studies showing beneficial roles of microglia-secreted factors in this context, reviewed in Miron (2017). In this respect, one of the most studied is insulin-like growth factor 1 (IGF-1). Interestingly, expression and release of IGF-1 were found to be restricted to a particular subset of microglia, characterized by the expression of the integrin CD11c, representing a consistent fraction of all microglial cells populating white matter regions during developmental myelination and in response to myelin injury (Wlodarczyk et al., 2017, 2021; Cao et al., 2021). Another important molecule involved in the pro-remyelinating properties of microglia is tumor necrosis factor (TNF), whose trans-membrane form (tmTNF) is known to promote trophic and regenerative responses by interacting with the receptor TNFR2 (Probert, 2015; Raffaele et al., 2020). TNFR2 activation on microglial cells was shown to regulate the expression and release of several neuroprotective factors, including granulocyte colony-stimulating factor (G-CSF), adrenomedullin, and IL-10 (Veroni et al., 2010). Accordingly, microglia-specific genetic ablation of TNFR2 exacerbated the pro-inflammatory activation of these cells, compromising their pro-regenerative functions, including tissue surveillance and phagocytosis (Gao et al., 2017). Consequently, knockout of microglial TNFR2 resulted in earlier disease onset and increased demyelination in an MS model (Gao et al., 2017). TNF released by microglial cells was also found to be required for the generation of new myelinating cells within demyelinated areas (Cunha et al., 2020). In detail, activation of the tmTNF/TNFR2 axis was shown to sustain OL maturation and remyelination, while TNFR2 ablation in OLs caused differentiation defects (Arnett et al., 2001; Madsen et al., 2016a). Hence, these results suggest that tmTNF/TNFR2 signaling may be required for the pro-remyelination properties of microglia, with important implications also in a stroke-related context. Indeed, conditional genetic ablation of solTNF with preservation of tmTNF resulted in reduced infarct volume and functional disability after MCAo (Madsen et al., 2016b). Microglia-dependent pro-myelinating effects have been also attributed to the expression of galectin-3 (Gal-3), a member of the family of β-galactoside-binding lectins (Thomas and Pasquini, 2018). Following myelin injury, expression of Gal-3 by microglia was found to favor a pro-regenerative microglial phenotype, fostering myelin debris phagocytosis through TREM2 activity (Hoyos et al., 2014). In addition, Gal-3 can be cleaved and released by microglia to directly interact with recruited OPCs in transition to the immature OL stage, enhancing their maturation (Pasquini et al., 2011).

Finally, another way by which microglia facilitate OPC-dependent remyelination is through extracellular matrix (ECM) remodeling (Lloyd and Miron, 2019). Indeed, also ECM molecules deposited into demyelinated ischemic lesions represent inhibitory cues hindering remyelination (Marangon et al., 2020). Microglia-derived matrix metalloproteinases (MMPs) were found to degrade fibronectin and chondroitin sulfate proteoglycans (CSPGs), which are known to inhibit OPC recruitment and differentiation (Pu et al., 2018; Wang et al., 2018). Another mechanism of microglia-mediated ECM modification is through the secretion of transglutaminase-2, which crosslinks laminin to regulate OPC proliferation and differentiation (Giera et al., 2018).

In general, the contribution of microglia to remyelination is to provide a favorable environment for myelin regeneration (Lloyd and Miron, 2019). Thus, a full understanding of the mechanisms underlying the pro-remyelinating properties of microglia (Fumagalli et al., 2018), and their communication with remyelinating OPCs could help design effective therapies supporting myelin repair.

## The Impact of Microglia-Derived Extracellular Vesicles on Oligodendrogenesis and Post-Stroke Remyelination

Previous studies have shown that blocking the activation of microglia by minocycline treatment, as well as depleting microglial cells through gadolinium chloride or CSF1R inhibitors, impairs the myelin repair process, suggesting the importance of the interaction between microglia and OLs during remyelination (Li et al., 2005; Tanaka et al., 2013; Pavic et al., 2021; Raffaele et al., 2021).

One of the means by which microglia communicate with other cells and exert their beneficial or harmful effects is the release of extracellular vesicles (EVs; Turola et al., 2012; Prada et al., 2013; Paolicelli et al., 2019). EVs are nanoparticles formed by a double layer of phospholipids resembling the cellular membrane, which can signal to adjacent cells or travel very long distances and deliver complex messages to distant cells (Van Niel et al., 2018). The cargo of EVs includes both lipophilic components present in the membrane fraction, including lipids and transmembrane proteins, and hydrophilic molecules retained in the cytosolic compartment, such as soluble proteins and nucleic acids, which are protected from enzymatic degradation by the vesicular membrane (Van Niel et al., 2018). EVs can be classified as microvesicles (MVs), originating from the plasma membrane, and exosomes, generated by the trafficking of multivesicular bodies (MVBs) from the cytosol to the plasma membrane (Basso and Bonetto, 2016). Since efficiently separating MVs from exosomes remains an unsolved technical challenge, the current classification distinguishes EVs based on their size rather than the mechanism of biogenesis, with large EVs being > 200 nm and small EVs < 100–200 nm (Théry et al., 2018).

The composition of microglial EVs was shown to reflect the activation state of the donor cell (Verderio et al., 2012; Garzetti et al., 2014). Microglial EVs profoundly influence the molecular signature and function of recipient cells by activating contact-mediated signaling pathways (Antonucci et al., 2012; Gabrielli et al., 2015) and/or transferring genetic information (Drago et al., 2017; Prada et al., 2018). Recently, microglial EVs have been identified as mediators of inflammation and neurodegeneration (Verderio et al., 2012; Delpech et al., 2019; Gabrielli et al., 2022). On the other hand, they have been also described as vehicles of pro-regenerative molecules, directing OLs towards maturation (Lombardi et al., 2019), and modulating the activation state of microglia residing in the damaged tissue, resuming their protective functions (Casella et al., 2018; Zhang et al., 2020). In this respect, infusion of pro-regenerative microglial EVs in the ipsilateral corpus callosum of ischemic mice was found to significantly increase OPC recruitment at lesion boundaries and enhance their maturation (Raffaele et al., 2021). These results were corroborated by another independent study, showing that intravenous delivery of pro-regenerative microglial EVs significantly promoted OL maturation and white matter structural remodeling after transient MCAo (Li et al., 2022b). In both cases, microglial EVs were able to successfully promote remyelination and long-term functional recovery after stroke (Raffaele et al., 2021; Li et al., 2022b).

Different components of the cargo of microglial EVs have been implicated in promoting OPC maturation. In this respect, the role of vesicular tmTNF in promoting OL differentiation has been demonstrated, as simultaneous treatment with the TNF inhibitor etanercept abolished the pro-differentiating effects of microglial EVs (Raffaele et al., 2021). Furthermore, the purified lipid fraction of microglial EVs was found to promote OPC maturation even more efficiently than intact EVs, although the precise vesicular lipids responsible for this effect remain to be elucidated (Lombardi et al., 2019; Gualerzi et al., 2021). Finally, the pro-differentiating effects of microglial EVs may be also partly mediated by their miRNA cargo, as the knockdown of miRNA-23a-5p in donor microglia was found to abolish the pro-myelinating properties of EVs (Li et al., 2022b).

In parallel, pro-regenerative microglia-derived EVs were also shown to significantly foster the pro-resolving properties of recipient pro-inflammatory and dystrophic microglial cells at the boundary of the ischemic lesion, contributing to create a permissive environment for remyelination (Raffaele et al., 2021; Zhang et al., 2021). On this basis, microglia-derived EVs represent potential regenerative tools to enhance functional recovery after stroke, by simultaneously targeting OLs and microglia around the ischemic lesion.

## Concluding Remarks

Current literature strongly suggests the importance of microglia-to-OPCs communication for efficient myelin repair. Therapeutic interventions capable, at the same time, of directly promoting OPC maturation and sustaining the pro-regenerative functions of microglia in the local microenvironment may therefore represent the best option to achieve functional recovery after stroke, as well as in other neurological diseases characterized by demyelination and detrimental neuroinflammation.

## Author Contributions

SR and MF: conception of idea, review of the literature, figure preparation, and manuscript writing and editing. All authors contributed to the article and approved the submitted version.

## Conflict of Interest

The authors declare that the research was conducted in the absence of any commercial or financial relationships that could be construed as a potential conflict of interest.

## Publisher’s Note

All claims expressed in this article are solely those of the authors and do not necessarily represent those of their affiliated organizations, or those of the publisher, the editors and the reviewers. Any product that may be evaluated in this article, or claim that may be made by its manufacturer, is not guaranteed or endorsed by the publisher.
